# Avian Metapneumovirus subtype B around Europe: a phylodynamic reconstruction

**DOI:** 10.1186/s13567-020-00817-6

**Published:** 2020-07-08

**Authors:** Giovanni Franzo, Matteo Legnardi, Giulia Mescolini, Claudia Maria Tucciarone, Caterina Lupini, Giulia Quaglia, Elena Catelli, Mattia Cecchinato

**Affiliations:** 1 Department of Animal Medicine, Production and Health, University of Padua, Legnaro, PD Italy; 2 Department of Veterinary Medical Sciences, University of Bologna, Ozzano dell’emilia, BO Italy

**Keywords:** Avian Metapneumovirus, Phylodynamic, Europe, Molecular epidemiology, Evolution

## Abstract

Avian Metapneumovirus (aMPV) has been recognized as a respiratory pathogen of turkey and chickens for a long time. Recently, a crescent awareness of aMPV, especially subtype B, clinical and economic impact has risen among European researchers and veterinarians. Nevertheless, the knowledge of its epidemiology and evolution is still limited. In the present study, the broadest available collection of partial G gene sequences obtained from European aMPV-B strains was analyzed using different phylodynamic and biostatistical approaches to reconstruct the viral spreading over time and the role of different hosts on its evolution. After aMPV-B introduction, approximatively in 1985 in France, the infection spread was relatively quick, involving the Western and Mediterranean Europe until the end of the 1990s, and then spreading westwards at the beginning of the new millennium, in parallel with an increase of viral population size. In the following period, a wider mixing among aMPV-B strains detected in eastern and western countries could be observed. Most of the within-country genetic heterogeneity was ascribable to single or few introduction events, followed by local circulation. This, combined with the high evolutionary rate herein demonstrated, led to the establishment of genetically and phenotypically different clusters among countries, which could affect the efficacy of natural or vaccine-induced immunity and should be accounted for when planning control measure implementation. On the contrary, while a significant strain exchange was proven among turkey, guinea fowl and chicken, no evidence of differential selective pressures or specific amino-acid mutations was observed, suggesting that no host adaptation is occurring.

## Introduction

Avian Metapenumovirus (aMPV) is a well-known pathogen affecting particularly turkeys and chickens, although also other avian species including guinea fowls [1]⁠, pheasants [2]⁠ and ducks [3] ⁠can be infected. aMPV has been associated with upper respiratory tract infections in turkeys and chickens, which can lead to relevant clinical signs and economic losses, especially in presence of secondary infections [4]⁠.

aMPV is an icosahedral, enveloped virus belonging to the family *Pneumoviridae*, genus *Metapneumovirus*, and is featured by a single-stranded negative-sense RNA genome approximately 15 kb-long encoding for 8 genes located in the following order: 3′-Nucleoprotein (N), Phosphoprotein (P), Matrix (M), Fusion (F), Matrix 2 (M2), Small hydrophobic (SH), attachment (G) and large polymerase (L)-5′ [5]⁠. While L and P are non-structural proteins involved in genome replication, the others code for the nucleocapsid, matrix and envelope structural proteins [5]⁠. Among those, the research has focused especially on the G protein, a glycoprotein involved in the viral attachment, and the F one, a fusion protein fundamental for the fusion of the viral envelope with the cell membrane. Unfortunately, extensive studies investigating the interaction of these proteins with the host receptors and immune response are largely lacking. Nevertheless, they are considered likely targets of the host immunity because of their location on the virus surface [6, 7]⁠. Particularly, preliminary studies have suggested the presence of T cell epitopes on the G protein and its directional evolution after vaccination introduction, supporting its immunological relevance [8]⁠. Moreover, the higher genetic heterogeneity of the G gene compared to others, including the F one, makes it suitable for molecular epidemiological studies and strain characterization and has promoted a more intensive sequencing activity over time.

After its first detection in South Africa in the late 1970s, aMPV and/or related syndromes were described in several European countries: the United Kingdom [9]⁠, France [10]⁠, Spain [9]⁠, Germany [11]⁠, Hungary [12]⁠ and Italy [13, 14]⁠. Since then, aMPV has been detected in most areas of the world where poultry are raised commercially [5]⁠. Initial serological assays based on monoclonal antibodies evidenced a certain variability among aMPV strains [15–17]⁠, which was then confirmed by genetic analysis [18]⁠, leading to the differentiation between aMPV subtype A and B. In the US, previously considered aMPV free, a new, highly divergent subtype (named aMPV-C) was detected in 1996 [19]⁠. Differently from subtype A and B, which show a worldwide distribution, including Europe, Africa, Asia and South America continents, aMPV-C circulates mainly in the US, even if it has been sporadically described in minor species (Muscovy duck and pheasant) in Korea, France and China [19–22]⁠. Its presence in Chinese commercial chickens has also been reported once [23]⁠. Finally, a retrospective study performed on strains isolated from turkeys in France in 1985 demonstrated the presence of a different subtype, designated subtype D [24]⁠, which has never been reported again.

Because of their broader distribution and high detection frequency, subtype A and B were long considered a relevant threat for the poultry industry. However, most recent epidemiological studies performed in Europe have consistently reported the absence of aMPV-A, which was clearly outclassed by aMPV-B [25–28]⁠.

Paradoxically, although those studies have demonstrated the high prevalence of this subtype and the concern among researchers and field veterinarians on its role as a primary pathogen is rising, our knowledge of aMPV-B molecular epidemiology, spreading patterns, population dynamics and evolutionary rate is still at its infancy.

Aiming to fill this gap, a phylodynamic analysis has been performed on the broadest available dataset of partial G gene sequences obtained from European aMPV-B strains.

## Materials and methods

### aMPV sequence dataset

All freely available European aMPV-B G gene sequences were downloaded from Genbank. Sequences were included in the study only if collection year and country were available. When known, the host species was recorded. No clusters of multiple sequences originating from the same outbreak were selected. Reference vaccine strains were also included.

All sequences were aligned at codon level and then back-translated to nucleotides using the MAFFT [29]⁠ method implemented in TranslatorX [30]⁠.

Since the presence of recombinant or vaccine strains can severely affect the topology reconstruction, population parameter estimation and obscure the temporal signal, these sequences were removed from the dataset.

The presence of recombination events was evaluated using GARD [31]⁠ method, implemented in Datamonkey. A preliminary phylogenetic tree was reconstructed using IQ-Tree [32]⁠, selecting as the best substitution model the one with the lowest Akaike Information Criteria, calculated by the software itself. Potential vaccine or vaccine-derived strains were removed based on a combination of the following criteria:

1. Strong clustering (bootstrap support > 70) with reference vaccines;
2. Percentage of identity higher than 99% compared to reference vaccines;
3. Evaluation of marker positions;
4. Expert opinion, evaluating the temporal relationship between vaccine commercialization or use and the collection year of the strain.

When available, data on vaccine administration, flock history and country-specific molecular epidemiology were taken into account for strain classification.

The refined alignment was trimmed to include the longest region still achieving a full coverage among the sequences. The list of sequences included in the final dataset is reported in Additional file 1.

The presence of an adequate phylogenetic signal was assessed by likelihood mapping analysis performed with IQ-TREE.

After strain selection, a new phylogenetic tree was generated, and TempEst was used to preliminarily evaluate the temporal signal of the aMPV-B phylogeny and therefore the applicability of molecular clock-based methods.

### Phylodynamic analysis

The time to the most recent common ancestor (tMRCA), substitution rates and population dynamics were jointly estimated using a Bayesian serial coalescent approach implemented in BEAST 1.8.4 [33]⁠.

The best substitution model was selected based on the Bayesian Information Criterion calculated using JmodelTest [34]⁠ while the molecular clock was selected by comparing the different models (strict vs relaxed molecular clock) based on Bayesian Factor (BF), which was calculated through estimation of the marginal likelihood of the different models using the path sampling (PS) and stepping stones (SS) methods [35]⁠. The non-parametric Skygrid model [36]⁠ was selected to infer viral past population dynamics (i.e. Effective population time × generation time; Ne × t).

The reconstruction of viral migration among countries was simultaneously performed using the discrete-trait phylogeographic approach described by Lemey et al. [37]⁠. A Bayesian stochastic search variable selection (BSSVS) was also implemented to allow the calculation of a BF test that identified the most parsimonious description of the spreading process.

A comparable ancestral discrete trait reconstruction, coupled with BSSVS, was used to estimate the viral flux among available hosts. The best migration model, i.e. symmetric vs asymmetric, was selected based on Bayesian Factor (BF), calculated through estimation of marginal likelihood of the different models (different combinations of host and country symmetric and asymmetric models were tested) using the PS and SS methods, as previously described. The more complex model was considered an improvement over the simpler one if the relative BF was higher than 5.

All parameters were estimated performing a 1 billion generation Markov Chain Monte Carlo run. Results were analyzed using Tracer 1.6 and accepted only if the estimated sample size (ESS) was greater than 200 and the convergence and mixing were adequate. After the exclusion of a burn-in equal to 20% of the run length, parameter estimation was summarized in terms of mean and 95% Highest Posterior Density (HPD). Maximum clade credibility (MCC) trees were constructed and annotated using Treeannotator (BEAST package).

SpreaD3 [38]⁠ was used to display the spreading process over time and to calculate the BF associated to each migration route. The transition rates among countries were considered statistically supported when the BF was greater than 5.

### Selective pressures

The action of selective pressures was compared between chicken and turkey (guinea fowl was excluded due to the limited sequence availability) using the dNdSDistributionComparison.bf implemented in HyPhy [39]⁠. Differences in the site-by-site selection patterns among different hosts were investigated using the batch files CompareSelectivePressure.bf implemented in the same program. The presence of episodic directional selection was also tested on the whole dataset with the MEDS method [40]⁠, marking the sequences collected in chickens as foreground branches.

## Results

### Dataset

Out of the 202 sequences initially included in the study, 71 were excluded since they were classified as likely vaccine or vaccine-derived strains. Therefore, the final dataset included 131 sequences, encompassing a region of 330 bp, originating from 9 countries (i.e. France, Greece, Italy, Romania, Russia, Spain, the Netherlands, Ukraine and the United Kingdom) in the period 1985–2019.

Despite the limited size of the considered region, the likelihood mapping analysis demonstrated the presence of an adequate phylogenetic signal. Similarly, TempEst investigation revealed that the positive correlation between genetic divergence and sampling time (i.e. R = 0.68) was high and suitable for phylogenetic molecular clock analysis.

### Phylodynamic analysis

**Figure 1:**
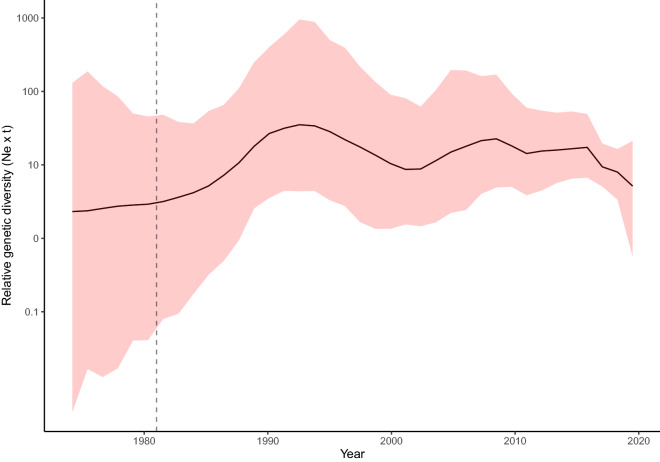
**Mean relative genetic diversity (Ne × t) of the European aMPV population over time.** The upper and lower 95HPD values are reported as shaded areas. The mean tMRCA is reported as a dotted vertical line.

**Figure 2:**
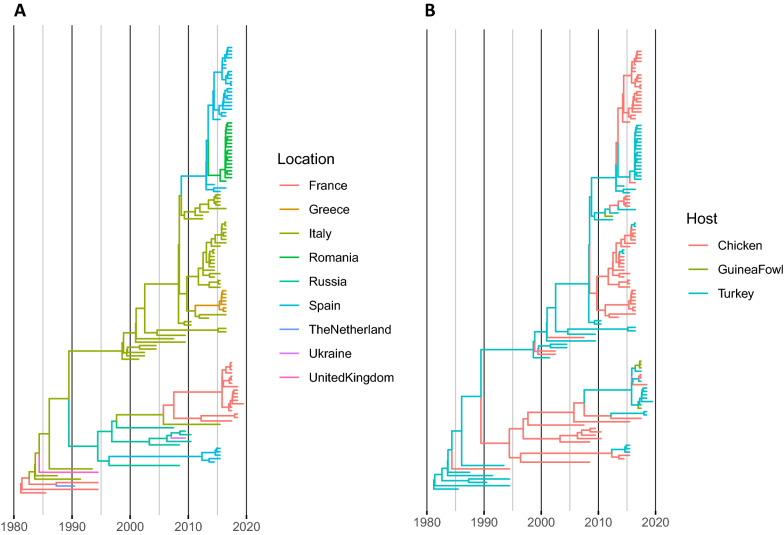
**Time calibrated phylogenetic trees.** The tree branches have been colour-coded according to the collection country (**A**) or host species (**B**) predicted with the highest posterior probability.

The tMRCA of European aMPV-B strain was estimated in 1981.17 (95HPD 1971.93–1985.28) and the evolutionary rate was 1.21 · 10^−3^ (7.11 · 10^−4^–1.83 · 10^−3^ substitutions per site per year). The Bayesian skygrid reconstruction of the relative genetic diversity evidenced, after the marked rise following aMPV-B introduction, a substantially constant viral population size until approximately 2015, when a certain decrease in population size was observed (Figure 1). A symmetric migration model was preferred over the asymmetric one based on BF calculation for both country and host species. The phylogeographic reconstruction demonstrated a tendency of aMPV-B strains to form mainly country-specific clusters, being single introduction events able to explain most of the genetic variability observed within country (Figure 2A and Additional file 2). Particularly, the first aMPV-B introduction was estimated to have occurred in France in 1981 (95HPD 1971.93–1985.28), followed by a migration to Italy few years later in 1984 (95HPD 1980.38–1986.98). These countries were the most likely source of further spreading to other European countries, including the Netherlands in 1987 (95HPD 1984.07–1989.73) and the United Kingdom in 1989 (95HPD 1987.33–1992.25), and then to Russia, in 1996 (95HPD 1989.71–2000.9). Russia was subsequently involved in the spreading to Ukraine in 2007 (95HPD 2003.62–2008.61), Spain in 2005 (95HPD 2000.97–2007.51) and also back to Italy in 2012 (95HPD 2008.97–2014.96). In the following years, Italy, in particular, was responsible for the introduction of new aMPV-B strains to Spain in 2011 (95HPD 2008.38–2011.18), Greece in 2012 (95HPD 2010.23–2012.99) and France in 2012 (95HPD 2008.97–2014.95). A late, potential connection, was also estimated between Spain and Romania in 2015 (95HPD 2012.38–2016.95) (Figure 2A and Additional file 2).

**Figure 3:**
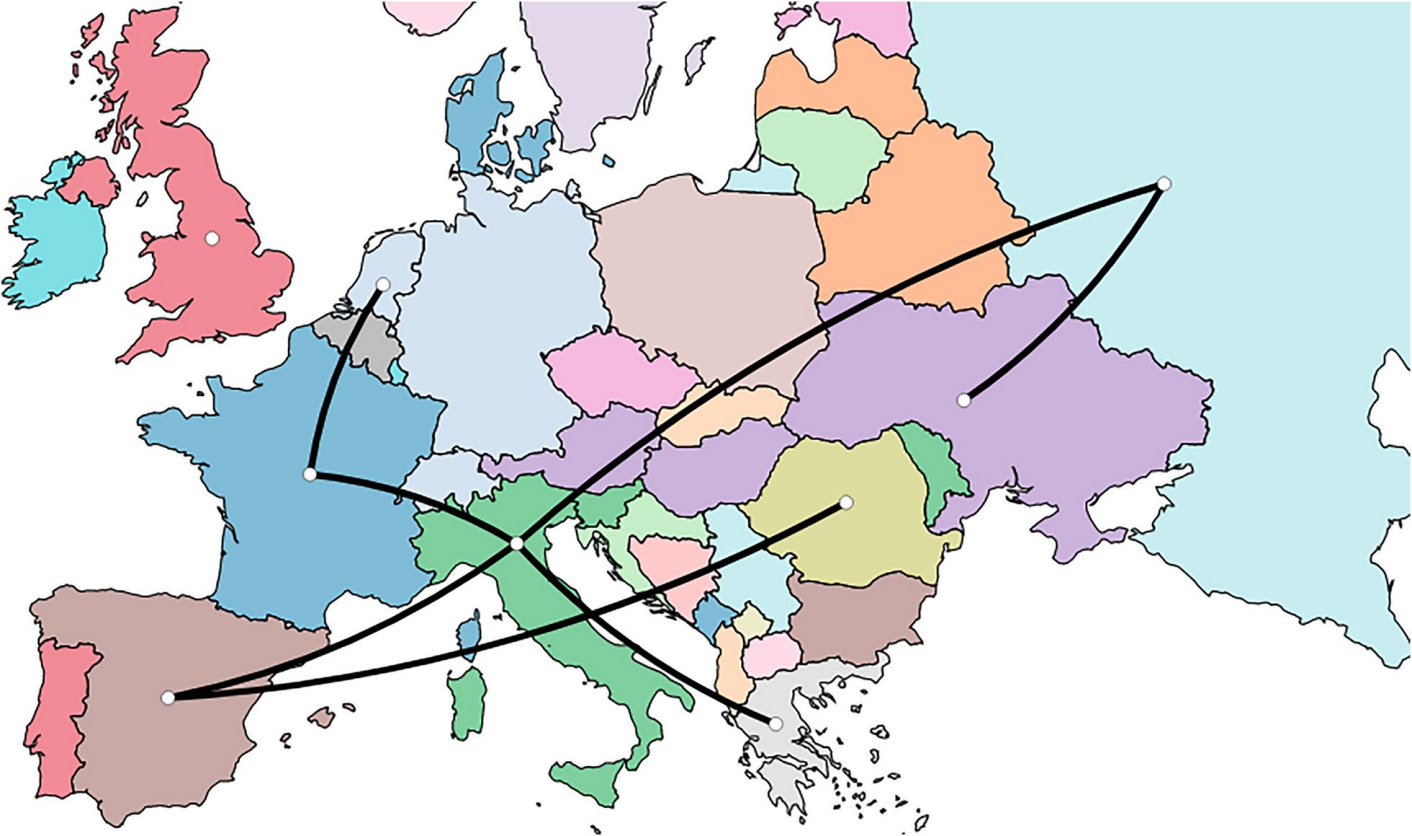
**Map reporting the well-supported migration paths (i.e. BF > 5) among European countries.**

However, only migration routes from France to Italy and the Netherlands, from Italy to Greece and Spain, from Spain to Romania and from Russia to Ukraine were well supported statistically (Figure 3).

When collection host species was evaluated, a certain tendency to host-specific clustering was displayed (Figure 2B). However, several exceptions were demonstrated, especially in countries like Italy where both species are reared in close proximity. Guinea fowl derived strains were part of three different clades, and the respective ancestors were always predicted to circulate in turkeys. Accordingly, statistically supported transmission routes were observed between chicken and turkey, and between turkey and guinea fowl.

### Selective pressures

**Figure 4:**
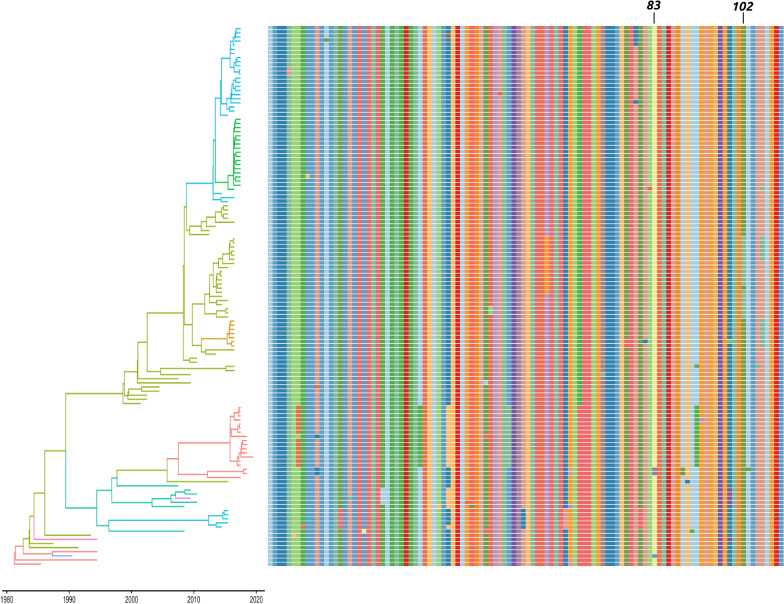
**Plot reporting the alignment of the amino acids of the partial G gene with respect to their position in the phylogenetic tree (strains have been colour-coded according to the collection country with the highest posterior probability).** Amino acid positions under diversifying and directional selection have been reported.

**Figure 5:**
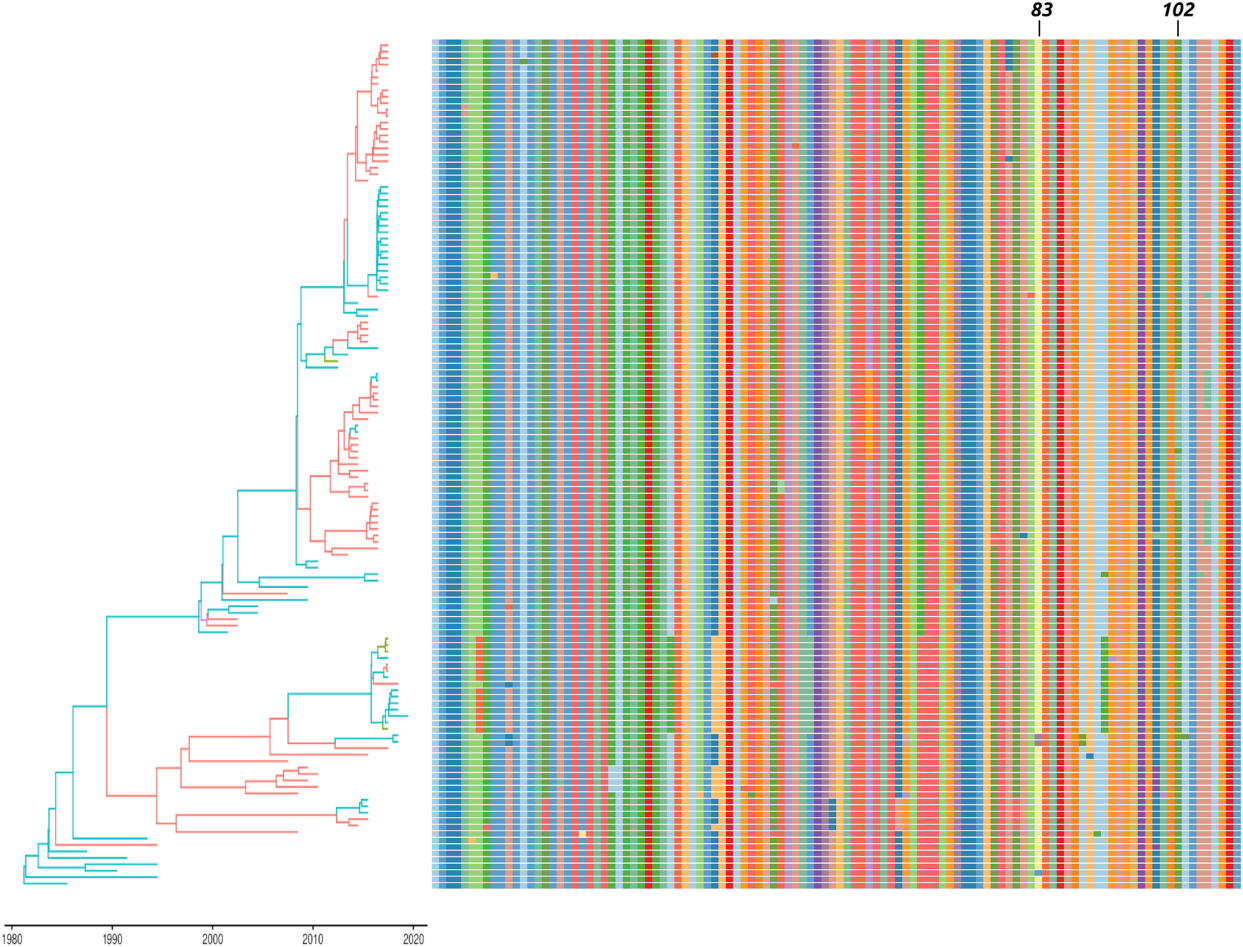
**Plot reporting the alignment of the amino acids of the partial G gene with respect to their position in the phylogenetic tree (strains have been colour-coded according to the collection host species with the highest posterior probability).** Amino acid positions under diversifying and directional selection have been reported.

The analysis of differential diversifying selection acting on strains collected from chickens and turkeys reported only one position, i.e. aa 83, where chicken collected strains appeared under a stronger selection. On the other hand, episodic directional selection acting on strains introduced in chickens affected only position 102, where a tendency to mutate toward alanine was observed (Figures 4 and 5).

However, when the action of selective forces, the proportion of selected sites and selective regimes (i.e. both proportion of involved sites and selective strength) were compared between the alignment of strains collected from chickens and turkeys, no statistically significant differences were detected.

## Discussion

Although aMPV is a relevant pathogen for the poultry industry in Europe, our knowledge of its molecular epidemiology is still remarkably poor and based on a limited number of studies performed by few research groups. The present study aims to provide a more comprehensive description of its epidemiology and evolution, based on a robust modelling and statistical approach.

Two initial obstacles had to be overcome. At first, the low number of freely available sequences confirms the limited aMPV sequencing activity of most European research groups. This scenario is further complicated by the lack of a consensus on the genomic region targeted for sequencing, thus creating poorly overlapping (and therefore incomparable) datasets. Nevertheless, the recent release of a relevant number of sequences (Mescolini et al., unpublished) collected from different countries over time allowed to increase the aMPV strain representativeness. Although the limited sample size and the relatively short analyzed genomic region surely represent unavoidable limits of the study, the analysis of the phylogenetic and temporal signal demonstrated that the dataset was informative enough to obtain reliable results. Secondly, the vast use of live attenuated vaccines able to circulate for a long time in the field, potentially even as revertant strains [41–43]⁠, complicates the differentiation of actual field strains from the vaccine or vaccine-derived ones. Since vaccine strains are continuously introduced in poultry farms but do not display any evolution (at least not before being administered to animals), the sequencing of the same vaccine strain year after year surely obscures and biases the temporal signal [44]⁠. The selected approach to remove vaccine-derived strains seemed effective since a high temporal signal could be proven in the refined sequence dataset. Especially, a relevant improvement in the correlation coefficient between genetic divergence and sampling time was demonstrated compared to the initial database (i.e. R = 0.68 vs R = 0.36).

Based on these results, the origin of aMPV-B could be reliably estimated. Interestingly, the tMRCA of aMPV-B in Europe was inferred in the 1981.17 (95HPD 1971.93–1985.28) in France, which closely fits with the earlier reports based on epidemiological evidence [10]⁠, suggesting at the same time a prompt identification of this emerging disease and the robustness of our estimates. Thereafter, the infection spread was relatively quick, involving initially Western and Mediterranean Europe until the end of the 1990s, and then spreading westwards at the beginning of the new millennium.

In the following period, a wider mixing among aMPV-B strains detected in eastern and western countries could be observed. France, Spain and particularly Italy seem to have played a major role in the aMPV-B spread in the last years. Explaining the reasons behind the observed scenario is challenging due to the limited available information. The rapid diffusion among European countries has been demonstrated for Infectious bronchitis virus (IBV) QX and Q1 genotypes [45, 46]⁠; it is therefore not unexpected for another respiratory virus like aMPV to follow similar pathways. The live poultry trade could have allowed the viral transmission over long distances and the creation of a European Single Market could have facilitated the process, together with the poultry industry intensification and globalization increasing the viral migration rate in the last decades. It must be stressed that the differential sequence availability, increasing over time, could also partially justify the observed pattern, allowing the model to detect more contacts among counties in recent years. The pivotal role of some countries like France and Italy in aMPV-B epidemiology could be linked to the higher number of turkeys, traditionally considered the main viral host species, historically reared in these countries. However, many recent studies have reported a high aMPV detection frequency also in chickens [25–28] and the present results demonstrate a relevant strain exchange between the two species, making unlikely the preeminent role of turkeys in aMPV epidemiology. Alternatively, the high proportion of sequences obtained from Italy and other countries could have led to an overestimation of their epidemiological role. Another fascinating hypothesis brings into play the wild birds in long distances aMPV spreading, as initially suspected to justify its introduction in the United Kingdom [47, 48]. Because of the presence of migratory flyways like the Black sea/Mediterranean flyway overflying both Mediterranean countries and Russia, wild species could be involved in the bidirectional viral migration between these regions. Unfortunately, there are no current clear pieces of evidence of aMPV-B presence in wild birds to support this hypothesis [49]⁠. Additionally, an alternative path involving intermediate steps in the viral dispersal could have been missed due to the absence of sequences compliant with our inclusion criteria (e.g. aMPV was reported in Hungary, Poland and Croatia in the middle of the 1990s) [5].⁠

Therefore, a more intense sampling activity both in turkeys and chickens, coupled with data sharing, would be of sure benefit to improve the understanding of the actual aMPV spreading patterns in Europe, aiding its control.

Despite this uncertainness, the tendency of aMPV to establish in a country and form a monophyletic, independent clade appears quite clear and few multiple introduction events were noted. Therefore, similarly to what reported for IBV, the introduction of a new strain is a relatively rare phenomenon [45, 50]⁠. However, the capacity of preventing its maintenance and spread within-country appears limited, suggesting the inefficacy of currently implemented biosecurity measures.

Of note, the estimated viral population size, after the rise occurred in the first years following its introduction, remained substantially stable, as expected for an endemic infection, until approximately 2015, when a certain contraction was estimated. The adoption of vaccination programs even in broilers in different countries could reasonably explain a lower viral circulation. This evidence is in contrast with the increase in aMPV relevance reported by field veterinarians. However, the perceived increase in aMPV detection frequency could be actually due to a more intensive diagnostic activity, rather than a true epidemiological change.

Although no formal studies had been performed, aMPV was traditionally considered a slowly evolving virus, especially compared to other RNA viruses affecting poultry. Our estimations, on the other hand, revealed a substitution rate fully within the range of the RNA viruses [45, 46, 51]. The high propensity to mutate could have been expected considering the presence of subpopulation even in vaccine strains [52]⁠, the easiness of those strains to undergo reversion to virulence [43]⁠, and the evidence of aMPV-B vaccine driven evolution in Italy [8, 53]⁠.

A non-negligible heterogeneity could be observed at the amino acid level also. As expected, members of the same clade tended to show similar amino acid mutations, although several exceptions were identified, potentially ascribable to both dead-end or-low-fitness variants or to new emerging ones. Because the clade structure was highly correlated with geographic clustering (Figure 4), it can be concluded that strains with different phenotypic features circulated in different countries. aMPV vaccines, although able to reduce disease occurrence and viral shedding, are not equally effective in preventing infection and viral circulation, probably contributing to the above-mentioned within-country viral persistence. If this phenotype variability affects vaccine efficacy should be carefully evaluated in order to allow the selection of the best vaccine based on the epidemiological situation.

On the other hand, no clear association was identified between amino acid sequence and host, at least in the considered region (Figure 5).

The analysis of directional selective pressure acting on strains circulating in chickens evidenced only one site, i.e. codon 102, under this kind of selection. Similarly, a differential diversifying selection was reported only for codon 83. However, in both cases, the phenotype variability was limited to a single clade and the contribution of other factors is therefore hard to be excluded. Moreover, when the action of selective forces, proportion of selected sites and selective regimes acting in the two hosts were compared, no statistically significant differences could be detected.

Taken as a whole, these results provide no evidence to claim a host-specific adaptation of aMPV-B strains. If other sites in the viral proteins allow for a higher fitness in chickens remains to be established.

The present study demonstrates that, after its first introduction, aMPV-B was able to rapidly spread in Western European countries and in the Eastern ones thereafter. Nevertheless, the molecular epidemiological scenario is determined mainly by single introduction events followed by independent rapid evolution. Since this has led to the presence of strains with different amino acid profiles in different countries, the efficacy of currently available vaccines should be carefully evaluated.

Although relevant, the obtained results are surely limited by the lack of data due to the scarce sequencing activity, poor standardization of the sequenced region (generating not comparable information) and reluctance to share data. All these issues should be significantly improved to allow a proper comprehension of aMPV epidemiology, posing the basis for a more effective control.

## Supplementary information

**Additional file 1. Summary of selected sequences and relative metadata.** The list of Genbank accession numbers of the sequences used in the present study is reported together with relevant metadata (i.e. strain name, collection country, host and year). When no accession number was available, the relative reference has been provided.

**Additional file 2. Phylogeographic reconstruction of aMPV-B spreading in Europe.** The spreading of aMPV-B is reconstructed over time. Migration events are represented as lines, colour-coded from black to red according to the time period. The size of the polygons around a sampling location is proportional to the number of lineages maintaining that location, thus capturing the absolute and relative intensity of the local virus spread at any given point in time.
