# Supplementary figures and images for: Avian Metapneumovirus subtype B around Europe: a phylodynamic reconstruction
Source: Vet Res. 2020 Jul 8;51:88. doi: 10.1186/s13567-020-00817-6 (PMC7346485; doi:10.1186/s13567-020-00817-6)

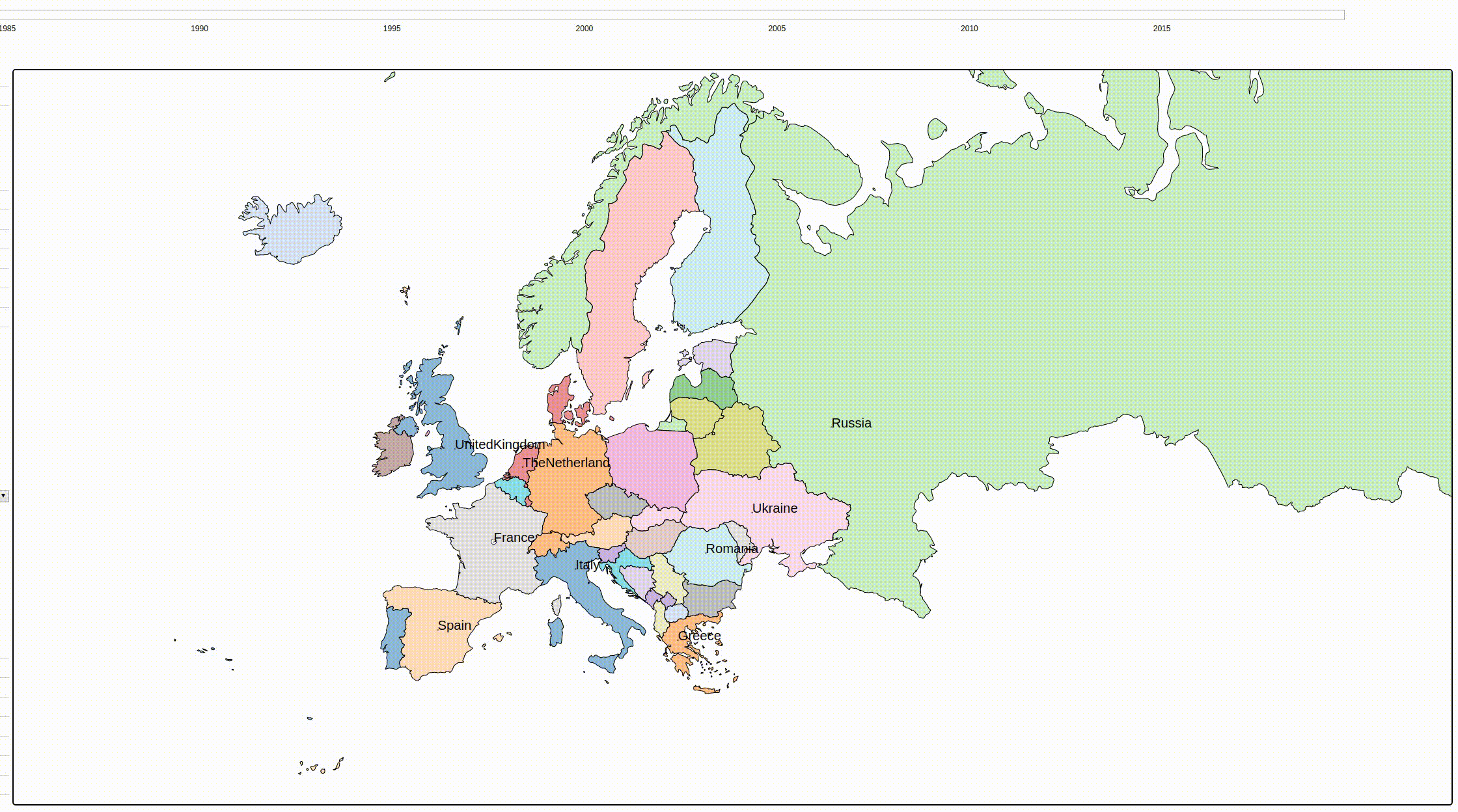

Supplement: Supplementary file 2 — Additional file 2. Phylogeographic reconstruction of aMPV-B spreading in Europe. The spreading of aMPV-B is reconstructed over time. Migration events are represented as lines, colour-coded from black to red according to the time period. The size of the polygons around a sampling location is proportional to the number of lineages maintaining that location, thus capturing the absolute and relative intensity of the local virus spread at any given point in time. [file 13567_2020_817_MOESM2_ESM.gif]
